# Genome-wide association study of Nelore and Angus heifers with low and high ovarian follicle counts

**DOI:** 10.1590/1984-3143-AR2023-0110

**Published:** 2024-02-05

**Authors:** Bárbara Loureiro, Ronaldo Luiz Ereno, Antônio Guilherme Roncada Pupulim, Maria Clara Viana Barroso Tramontana, Henrique Passos Tabosa, Ciro Moraes Barros, Maurício Gomes Favoreto

**Affiliations:** 1 Laboratório de Fisiologia da Reprodução Animal, Universidade Vila Velha - UVV, Vila Velha, ES, Brasil; 2 Departamento de Farmacologia, Instituto de Biociências, Universidade Estadual Paulista - UNESP, Botucatu, SP, Brasil

**Keywords:** Bovine HD 770 K SNP, candidate genes, follicle, reproduction, SNPs

## Abstract

The number of antral follicles is considered an important fertility trait because animals with a high follicle count (HFC) produce more oocytes and embryos per cycle. Identification of these animals by genetic markers such as single nucleotide polymorphisms (SNPs) can accelerate selection of future generations. The aim of this study was to perform a genome wide association study (GWAS) on Nelore and Angus heifers with HFC and low (LFC) antral follicle counts. The groups HFC and LFC for genotyping were formed based on the average of total follicles (≥ 3 mm) counted in each breed consistently ± standard deviation. A total of 72 Nelore heifers (32 HFC and 40 LFC) and 48 Angus heifers (21 HFC and 27 LFC) were selected and the DNA was extracted from blood and hair bulb. Genotyping was done using the Illumina Bovine HD 770K BeadChip. The GWAS analysis showed 181 and 201 SNPs with genotype/phenotype association (P ≤ 0.01) in Nelore and Angus heifers, respectively. Functional enrichment analysis was performed on candidate genes that were associated with SNPs. A total of 97 genes were associated to the 181 SNPs in the Nelore heifers and the functional analysis identified genes (ROBO1 and SLIT3) in the ROBO-SLIT pathway that can be involved in the control of germ cell migration in the ovary as it is involved in lutheal cell migration and fetal ovary development. In the Angus heifers, 57 genes were associated with the 201 SNPs, highlighting Fribilin 1 (FBN1) gene, involved in regulation of growth factors directly involved in follicle activation and development. In summary, GWAS for Nelore and Angus heifers showed SNPs associated with higher follicle count phenotype. Furthermore, these findings offer valuable insights for the further investigation of potential mechanism involved in follicle formation and development, important for breeding programs for both breeds.

## Introduction

Reproductive performance is an important trait in livestock production. The number of antral follicles (AFC) greater than 3 mm has been associated with fertility in dairy cows. Dairy cows with low follicle counts have lower pregnancy rates at first service, a longer interval from calving to conception, and a higher number of services during the breeding season compared to cows with high follicle counts (HFC; Mossa et al., 2012). In addition, cows with a HFC have a higher number of total oocyte recovery by ultrasound-guided follicular aspiration (OPU) and consequently a greater number of viable embryos produced in vitro (Ireland et al., 2007).

Indicine animals (Brahman and Nelore) have been shown to have a greater number of antral follicles, whereas taurine animals (Angus) have a greater number of preantral follicles (Cushman et al., 2019; Favoreto et al., 2019). In addition, a microarray study showed that the gene expression profile differs between these breeds. Several genes associate with osteoblast differentiation (BMP4, IGF1, IGFBP3 and IGFBP5), cell proliferation (BMP4, SATB1, EMX2, IGF1, MAP7, EMPEP and FABP7) and bone development (BMP4, COL13A1, IGF1, IGFBP3 and IGFBP5) showed higher expression in follicles of Nelore heifers. The genes are related to follicle activation and development (Favoreto et al., 2019). Moreover, genomic studies have shown that Indian and Taurine cattle differ in the expression of genes associated with important traits (Liu et al., 2021), SNPs (Dar et al., 2021; Verardo et al., 2021), gene ontologies (Cortez et al., 2022), and chromatin and methylation profiles (Powell et al., 2023; Capra et al., 2023). Even though there are studies showing that zebu and taurine cattle trace back to a common ancestor, Bos primigenius (Perez-Pardal et al., 2010; Pitt et al., 2018).

In the past years, the use of genomic information has been widely employed as a strategy to improve selection for phenotypes such as increased milk production (Chamberlain et al., 2012), fertility (Huang et al., 2010; Peñagaricano et al., 2012), and other traits of economic interest in cattle. Genome-wide association studies (GWAS) are a method in genetics that aims to identify specific genetic variations, known as single nucleotide polymorphisms (SNPs). The studies are made based on a population that has a specific trait, these animals will have their genotype searched for a common SNPs that can be associated with the phenotype (Dehghan, 2018). Single nucleotide polymorphisms (SNP) enabled the identification of a subset of markers that can explain important portions of the variation in these traits. An alternative approach to identifying SNPs associated with a phenotype of interest is a candidate gene approach, in which individual genes are selected as candidates based on their known function (Naukkarinen et al., 2010).

Identification of SNPs and possible genes responsible for genetic variation in Nelore and Angus with HFC and LFC may improve understanding of the biological pathways involved in the AFC phenotype.

In summary, animals that are considered HFC are associated with better fertility as they allow more oocytes per cycle and thus more embryos in in vivo and in vitro embryo production (Ireland et al., 2007). The identification of these animals by genetic markers could improve the fertility of future generations. Therefore, the aim of the present work was to perform a GWAS study in Nelore and Angus heifers with HFC and LFC using the high-density SNP array to identify genetic variants and possible genes associated with these phenotypes. The main hypothesis was that HFC animals have genetic markers associated with follicular development that could be used as biomarkers for genomic selection.

## Material and methods

### Animals selection and phenotypic data

Animals were maintained according to the Bioethics Committee of the Faculty of Veterinary Medicine (N° 439) and Animal Sciences of the São Paulo State University (Botucatu, São Paulo, Brazil) and in accordance with the specific guidelines and standards of the SSR.

To pre-select animals, a group of 155 Nelore and 132 Angus heifers of similar body weight (Nelore = 442.93 ± 6.97 kg and Angus = 466.23 ± 10,13) and age (25.57 ± 2,05) were examined by ultrasound (US; Mindray Vet DPS 2200, São Paulo, Brazil) with a 7.5-MHz probe on a random day of the estrous cycle. Only heifers that were cyclic and did not have a follicle greater than 5 mm were selected. In this initial evaluation, the total number of follicles ≥ 3 mm in both ovaries was counted and the mean follicle number for each breed was determined. These heifers were injected with two doses of PGF2 alpha 11 days apart to initiate luteolysis and synchronize the occurrence of ovulation. The ovaries of each animal were examined one day after ovulation in three consecutive estrous cycles to determine the high (HFC) and low (LFC) follicle count groups. Groups were formed based on the average of total follicles counted (≥ 3 mm) for each breed consistently ± standard deviation (Table 1). A total of 72 Nelore heifers were selected and classified into the groups: 32 animals were classified as HFC (40 ≥ follicles) and 40 animals were classified as LHC (20 ≤ follicles). For Angus heifers, 48 animals were selected and assigned to the groups: 21 animals were classified as HFC (20 ≥ follicles) and 27 as LFC (≤ 10 follicles).

**Table 1:** Numbers of animals, follicle average count and standard deviation for Nelore and Angus heifers with HFC and LFC.

	**Nelore**			**Angus**	
Number of animals	43	35		22	27
Fol average count	49	15		25	6
Standard deviation	9.3	3.9		5.1	2.3

### Sample and DNA extraction

During ultrasound examination a sample of blood from the tail vein or capillary bulbs from the tail hair were collected from all selected animals for DNA extraction. For the blood samples, DNA was extracted using the MiniPrep kit (Axygen bioscience, Union City, New Jersey, USA), while the capillary bulb was extracted using the NucleoSpin Tissue Kit (Macherey-Nagel, Duren, Germany) according to the manufacturer’s instructions. After extraction, the quality of the DNA samples was assessed by determining the A260/280 ratio in a biophotometer. Samples were accepted if the values were between 1.8 and 2.0. DNA was quantified and diluted to a concentration between 50 ng/µL and 150 ng/µL for subsequent genotyping.

### Genotyping and SNPs quality control

Genotyping was performed by DEOXI BIOTECNOLGIA LTDA, Araçatuba, São Paulo, Brazil, using the Illumina Bovine HD 770 K BeadChip (Infinium BeadChip, Illumina, San Diego, CA) according to the protocol of the manufacturer’s instructions. Genotypes were determined in Illumina A/B allele format and used to represent a covariate value at each locus coded as 0, 1, or 2 indicating the number of B alleles. Initial data analysis and visualization was performed using GenomeStudio Data Analysis Software (Illumina, 2023). For GWAS, SNPs were quality controlled using only autosomal SNPs with known genomic coordinates according to UMD 3.1 Bovine Genome. Samples with a call rate (IDCR) of less than 90% were removed from the study. SNPs were removed if they had a minor allele frequency ≤ 0.02, a call rate ≤ 0.98, and a P value for the Fisher exact test for Hardy-Weindberg equilibrium ≤ 1 x 0.00001. After filtering, 538.575 SNPs were used for the GWAS study.

### GWAS test and gene enrichment analysis

The Cochran-Armitage test has been used for genomic association between HFC and LFC in Nelore and Angus heifers. This test is commonly used as a genotype-based test for candidate gene association. It uses a range of scores that can be obtained as an efficient score test for logistic regression (Clarke et al., 2011). In this analysis, it was used for comparisons as a case-control study (GenABEL package; Aulchenko et al., 2007) with LFC as the control group in both subspecies. Significant SNPs (P ≤ 0.001) were mapped to corresponding or nearby genes by linkage disequilibrium (LD) using the software PLINK (Bush et al., 2009; Bohmanova et al., 2010). We considered only the genes with r^2^ > 0.8 significant for functional analysis. The genes associated with SNPs directly or indirectly by linkage disequilibrium were subjected to the functional enrichment analyzes on DAVID (Database for Annotation, Visualization and Integrated Discovery; (Dennis et al., 2003) to determine gene ontologies (GO), functions, and pathways in which the genes were overrepresented at P < 0.05.

## Results

**Figure 1:**
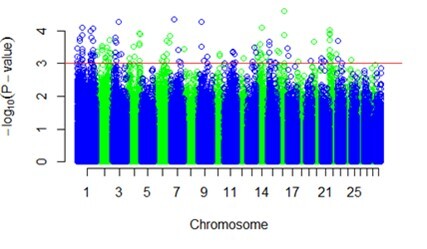
Genome-wide association analysis for HFC comparing with LFC in Nelore heifers. The Manhattan plot demonstrates the results of association after correction for population structure. The horizontal red line indicates the whole-genome with significance threshold [-log (p ≤ 10E-3)].

**Figure 2:**
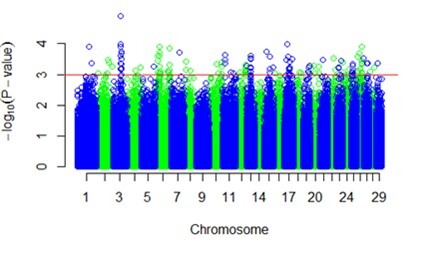
Genome-wide association analysis for HFC comparing with LFC in Angus heifers. The Manhattan plot demonstrates the results of association after correction for population structure. The horizontal red line indicates the whole-genome with significance threshold [-log (p ≤ 10E-3)].

After quality control, a total of 538.575 SNPs distributed on 29 chromosomes were used to study the association between phenotype and genotype. The profiles of p-values (in terms of -log[p]) of all tested SNPs for Nelore and Angus heifers are shown in Figures 1 and 2. A total of 181 SNPs for Nelore and 201 SNPs for Angus heifers showed an association (p ≤ 0.001) between the HFC and LFC groups.

The 181 SNPs of Nelore heifers were mapped on 23 different chromosomes, and the most significant SNPs (p ≤ 0.00098) were located on chromosomes 1, 3, 7, 14, 16, and 22 (Table 2). In Angus heifers, all 201 were mapped on 29 chromosomes, and the most significant SNP (p = 0.000124) is located on chromosome 3 (Table 2).

**Table 2:** Description of the most significant SNPs for Nelore e Angus heifers.

**Chromosome**		**SNPs**	
1	rs136289764 (8.11E-04)		
1	rs109443367 (8.47E-04)		
3	rs43704025 (5.39E-04)		rs43338364 (1.24E-04)
7	rs110807077 (4.53E-04)		
9	rs136692332 (5.39E-04)		
14	rs132707253 (8.01E-04)		
14	rs41730052 (8.01E-04)		
14	rs110253276 (8.01E-04)		
16	rs109100442 (2.47E-04)		
22	rs133905094 (9.38E-04)		

SNPs Identification: see Ensembl (2023).

GWAS revealed a total of 97 different genes associated directly or indirectly via LD (r^2^> 0.8) with all 181 SNPs of Nelore heifers (Table 3). Functional enrichment analysis was applied to all 97 genes associated in the integrated network using the DAVID Functional Annotation Tool. In Nelore heifers, a total of 5 gene ontologies (GO) were significantly overrepresented in 2 categories: biological process and cellular component (Table 5). In Angus heifers, 52 genes were associated with all 201 SNPs (Table 4), and functional enrichment analysis showed that 18 genes were significantly GO (p < 0.05) overrepresented in the 3 categories (biological process, cellular component, and molecular function; Table 5).

**Table 3:** Associated SNPs with respective candidate genes for Nelore heifers.

**Gene**	**Chromosome**	**SNP**	**Gene**	**Chromosome**	**SNP**	**Gene**	**Chromosome**	**SNP**
RSRC1	1	BTB-01568926	DC1I1	4	BovineHD0400003849	SGK3	14	Hapmap49131-BTA-34531
PLCH1	1	BovineHD0100031992	AGMO	4	BovineHD0400007019	PREX2	14	BovineHD1400009858
ANKUB1	1	BovineHD0100033727	A0JNG1	4	BovineHD0400001266	KCNB2	14	BTA-107899-no-rs
WWTR1	1	BovineHD0100033813	RERG	5	BovineHD0500027036	JPH1	14	BovineHD1400011391
AT1B3	1	BovineHD0100036157	BST1	6	BovineHD0600032820	SNTB1	14	BovineHD1400023903
EPHB1	1	BovineHD0100038454	NPNT	6	BovineHD0600005674	ZC3H12C	15	BovineHD1500005193
CEP63	1	BTB-00063883	A0JN38	6	BovineHD0600019557	F1N2Z9	15	BovineHD1500005842
APC13	1	BovineHD0100038705	EPHA5	6	BovineHD0600022781	E1BA24	15	BovineHD1500009535
ENTK	1	BovineHD0100005454	ART3	6	BovineHD0600025443	STIM1	15	BTA-114838-no-rs
C21orf63	1	BovineHD0100000686	11-Sep	6	BovineHD0600025874	LOC790886	16	BovineHD1600001673
ROBO1	1	BovineHD0100007707	FRAS1	6	BovineHD0600026271	RALGPS2	16	BovineHD1600017259
E1BJS9	1	BovineHD0100010534	SGCD	7	BovineHD0700020503	ANGL1	16	BovineHD1600017298
A5PKG1	1	BovineHD0100022104	HEM2	8	BovineHD0800031076	FBXW8	17	BovineHD1700017212
SMARCAL1	2	BovineHD0200030269	CLCN3	8	BovineHD0800000483	ADAMTS18	18	BovineHD1800001383
F1MTX0	2	BovineHD0200040258	TMEM2	8	BovineHD0800014467	F1MX91	18	ARS-BFGL-NGS-59215
KIAA1486	2	BovineHD0200033024	RIMS1	9	BovineHD0900002983	DOCK2	20	BovineHD2000000529
NMI	2	BovineHD0200013025	ZNF292	9	Hapmap54718-rs29022960	RNF180	20	BovineHD2000021261
MYO7B	2	BovineHD0200001364	EML5	10	BovineHD1000029437	FAM196B	20	BovineHD2000000555
IWS1	2	BovineHD0200001420	A6QLI2	10	BTA-114684-no-rs	SLIT3	20	BovineHD2000000133
DNAH7	2	BovineHD0200024162	A7YWN4	11	ARS-BFGL-NGS-10436	F1MJV4	20	BovineHD2000018883
ORC2	2	BovineHD0200025582	LBH	11	BovineHD1100019791	CCDC33	21	BovineHD2100021281
NRP2	2	BovineHD0200027119	FAM179A	11	BovineHD1100020297	LYZL4	22	BovineHD2200004477
PIK3R3	3	BTA-20822-no-rs	IFT172	11	BovineHD1100020629	F2Z4H7	22	BovineHD2200005456
NHRF3	3	BovineHD0300006834	Q3SZR7	11	BovineHD1100022452	AZI2	22	BovineHD2200000741
SYWM	3	BovineHD0300007564	HS1BP3	11	BovineHD4100009043	ZCWPW2	22	BovineHD4100015404
TBX15	3	BovineHD0300007604	XRN2	13	BovineHD1300011855	RBMS3	22	BTB-00830411
SPAG17	3	BovineHD0300007956	C20orf123	13	BovineHD1300021958	ATP2B2	22	ARS-BFGL-NGS-14331
MAN1A2	3	BovineHD0300035509	KIAA0146	14	BovineHD1400005988	EGFR	22	BovineHD2200000246
ATP1A1	3	BovineHD0300036028	PRKDC	14	BovineHD1400006081	DCDC2	23	BovineHD2300009744
Q2KI63	3	BovineHD0300002619	A6QLA9	14	BovineHD4100011332	TTC39C	24	BTB-01623856
ECHD2	3	BovineHD0300027093	A4IFV2	14	BovineHD4100011399	RB27B	24	BovineHD2400015606
AT1A2	3	BovineHD0300003116	TRIM55	14	BovineHD1400009278	CTNNA3	28	BTB-00980953
F1MY54	4	BovineHD0400033064						

**Table 5:** Gene Ontology terms related to biological process, cellular component and molecular function of the genes associated directly or indirectly with SNPs in the HFC from Nelore and Angus heifers.

	**Category**	**GO ~ Term**	**Count**	**%**	**P Value**	**Genes**
Nelore	GOTERM_BP_FAT	GO:0006928 ~ cell motion	5	11.4	0.02	ROBO1, PRKDC, DCDC2, DNAH7, SLIT3
	GOTERM_BP_FAT	GO:0048870 ~ cell motility	4	9.1	0.03	ROBO1, PRKDC, DCDC2, DNAH7
	GOTERM_BP_FAT	GO:0051674 ~ localization of cell	4	9.1	0.03	ROBO1, PRKDC, DCDC2, DNAH7
	GOTERM_CC_FAT	GO:0044459 ~ plasma membrane part	11	25.0	0.01	EPHA5, ART3, CLCN3, ROBO1, KCNB2, SNTB1, SGCD, STIM1, ATP1A1, RIMS1, CTNNA3
	GOTERM_CC_FAT	GO:0016010 ~ dystrophin-associated glycoprotein complex	2	4.5	0.03	SNTB1, SGCD
Angus	GOTERM_BP_FAT	GO:0001822 ~ kidney development	3	8.3	0.01	PKHD1, FBN1, NID1
	GOTERM_BP_FAT	GO:0001655 ~ urogenital system development	3	8.3	0.02	PKHD1, FBN1, NID1
	GOTERM_CC_FAT	GO:0043228 ~ non-membrane-bounded organelle	12	33.3	0.00	FNTB, TNS3, PARN, PKHD1, MYO16, RBM19, WDR1, MYH14, DNAH2, SHANK1, XRN2, CBS
	GOTERM_CC_FAT	GO:0043232 ~ intracellular non-membrane-bounded organelle	12	33.3	0.00	FNTB, TNS3, PARN, PKHD1, MYO16, RBM19, WDR1, MYH14, DNAH2, SHANK1, XRN2, CBS
	GOTERM_CC_FAT	GO:0005604 ~ basement membrane	3	8.3	0.01	FRAS1, FBN1, NID1
	GOTERM_CC_FAT	GO:0044420 ~ extracellular matrix part	3	8.3	0.02	FRAS1, FBN1, NID1
	GOTERM_CC_FAT	GO:0044430 ~ cytoskeletal part	6	16.7	0.03	FNTB, PKHD1, MYO16, MYH14, DNAH2, SHANK1
	GOTERM_CC_FAT	GO:0005730 ~ nucleolus	5	13.9	0.04	TNS3, PARN, RBM19, XRN2, CBS
	GOTERM_CC_FAT	GO:0005856 ~ cytoskeleton	7	19.4	0.04	FNTB, PKHD1, MYO16, WDR1, MYH14, DNAH2, SHANK1
	GOTERM_MF_FAT	GO:0004532 ~ exoribonuclease activity	2	5.6	0.02	PARN, XRN2
	GOTERM_MF_FAT	GO:0016896 ~ exoribonuclease activity, producing 5'-phosphomonoesters	2	5.6	0.02	PARN, XRN2
	GOTERM_MF_FAT	GO:0003779 ~ actin binding	4	11.1	0.02	MYO16, WDR1, MYH14, MYLK
	GOTERM_MF_FAT	GO:0005516 ~ calmodulin binding	3	8.3	0.03	ADCY1, MYH14, MYLK
	GOTERM_MF_FAT	GO:0003774 ~ motor activity	3	8.3	0.03	MYO16, MYH14, DNAH2
	GOTERM_MF_FAT	GO:0016796 ~ exonuclease activity, active with either ribo- or deoxyribonucleic acids and producing 5'-phosphomonoesters	2	5.6	0.03	PARN, XRN2
	GOTERM_MF_FAT	GO:0046872 ~ metal ion binding	14	38.9	0.04	FRAS1, ADCY1, RBM20, FBN1, SYT9, PRKCH, NID1, FNTB, TNS3, PARN, EBF1, XRN2, MYLK, CBS
	GOTERM_MF_FAT	GO:0043169 ~ cation binding	14	38.9	0.04	FRAS1, ADCY1, RBM20, FBN1, SYT9, PRKCH, NID1, FNTB, TNS3, PARN, EBF1, XRN2, MYLK, CBS
	GOTERM_MF_FAT	GO:0043167 ~ ion binding	14	38.9	0.04	FRAS1, ADCY1, RBM20, FBN1, SYT9, PRKCH, NID1, FNTB, TNS3, PARN, EBF1, XRN2, MYLK, CBS

**Table 4:** Associated SNPs with respective candidate genes for Angus heifers.

**Gene**	**Chromosome**	**SNP**	**Gene**	**Chromosome**	**SNP**	**Gene**	**Chromosome**	**SNP**
Q32KQ4	1	BovineHD0100028647	FBN1	10	BovineHD1000017884	SHANK1	18	ARS-BFGL-NGS-25117
CBS	1	BovineHD0100041801	PRKCH	10	BovineHD1000021003	DNAH2	19	BovineHD1900008265
MYLK	1	BovineHD0100019384	FNTB	10	BovineHD1000022127	LEPREL4	19	BovineHD1900012163
A8E661	2	BovineHD0200026032	A8E641	11	BovineHD1100020762	PSA4	21	ARS-BFGL-NGS-24797
PALMD	3	BovineHD0300013319	MYO16	12	BovineHD1200025736	PTPRG	22	BovineHD2200011275
IFI44	3	BovineHD0300019648	RALGAPA2	13	BovineHD1300011643	ATG7	22	BovineHD2200016071
KAD5	3	BovineHD0300019991	KIZ	13	BovineHD1300011819	CRISP1	23	BovineHD2300005886
ACM2	4	BovineHD0400028468	XRN2	13	BovineHD1300011851	PKHD1	23	BovineHD2300006404
TNS3	4	BovineHD0400020885	A6QQD3	15	BTB-01820462	FAM59A	24	BovineHD2400006778
ADCY1	4	BovineHD0400021263	SYT9	15	BovineHD1500013002	TNFRSF11A	24	BovineHD2400017755
F19A5	5	BovineHD0500034919	A6QNL3	16	BovineHD1600012798	PARN	25	BovineHD2500003745
PPP2R2C	6	BovineHD0600029214	F262	16	BovineHD1600001371	LIPA	26	BTA-62081-no-rs
SLC2A9	6	BovineHD0600030984	CD045	17	BovineHD1700011280	SORCS1	26	BovineHD2600007572
WDR1	6	BovineHD0600031016	CUX2	17	BovineHD1700016280	RBM20	26	BovineHD2600008410
A7YWG6	6	BovineHD0600008622	RBM19	17	BovineHD1700017987	F1MX91	18	ARS-BFGL-NGS-59215
FRAS1	6	BovineHD0600026329	LRBA	17	BovineHD1700002101	DOCK2	20	BovineHD2000000529
EBF1	7	BovineHD0700021323	MYH14	18	ARS-BFGL-NGS-3584	RNF180	20	BovineHD2000021261
A1A4J5	8	ARS-BFGL-NGS-1787				FAM196B	20	BovineHD2000000555

## Discussion

Genome-wide association studies (GWAS) were conducted to identify quantitative trait loci (QTL) and candidate genes associated with fertility in cattle. The most common traits that affect reproduction and are candidates for genetic selection are age at first calving (Hutchison et al., 2017; Mota et al., 2020), conception rate (Galvão et al., 2013), and daughter pregnancy rate (Parker Gaddis et al., 2014).

In Nelore and Angus heifers, the GWAS study showed no genomic region associated with variation in antral follicle number across all 29 autosomal chromosomes, which is shown in the Manhattan plots (Figures 1 and 2). The mean/low peaks observed in the figures can be attributed to two factors: Many SNPs have a small effect on phenotype and variable antral follicle number in cattle is regulated by many genes. GWAS identified several SNPs with association and candidate genes in animals with HFC in Nelore and Angus heifers when analyzed separately.

In Nelore heifers, the epidermal growth factor receptor (EGFR) gene was associated with the SNP BovineHD2200000246 on chromosome 22 (Table 3). EGFR is a transmembrane glycoprotein consisting of an extracellular ligand-binding domain and a cytoplasmic segment with tyrosine kynase activity, which is central to the cell proliferative effects of EGF. EGF has been shown to play a role in spermatogenesis, oocyte development and maturation (Wald, 2005; Richani and Gilchrist, 2018). In spermatogenesis, EGF enhances the effect of gonadotropin on testicular testosterone production by modulating the activity of enzymes involved in the biosynthetic pathway of testosterone and by increasing the availability of cholesterol for mitochondrial steroidogenesis (Wald, 2005). In follicular development, the EGF network is an essential component of the ovulatory cascade by relaying the signal LH from the periphery of the follicle to the cumulus-oocyte complex (COC). Although the EGF network act in the late stages of follicle development, a new concept to emerge is that cumulus cell acquisition of EGF receptor responsiveness in early stages of development represents a developmental hallmark in folliculogenesis. (Richani and Gilchrist, 2018; Ritter et al., 2015).

Nominal associated gene analyzes using DAVID bioinformatics resources identified several functional categories that differed in the HFC group compared with the LFC group in Nelore and Angus heifers. Notably, cell motion (ROBO1, PRKDC, DCDC2, DNAH7, and SLIT3), cell motility (ROBO1, PRKDC, DCDC2, and DNAH7), and localization (ROBO1, PRKDC, DCDC2, and DNAH7) were the GO associated with biological process in Nelore heifers. The Roundabout (ROBO) transmembrane proteins constitute a conserved family of receptors that includes ROBO1, ROBO2, ROBO3, and ROBO4, which together with their repellent ligand SLIT (SLIT1, SLIT2, and SLIT3) play an important role in regulating axon guidance decisions (Devine and Key, 2008). However, there is evidence that the SLIT/ROBO pathway also plays a role in cellular processes outside the nervous system (Wong et al., 2002). In a study of adult human ovaries, the expression of SLIT2, SLIT3, and ROBO2 was increased during the luteal phase and was negatively regulated by hCG and cortisol (Dickinson et al., 2008). In addition, blocking the activity of SLIT-ROBO decreased apoptosis and increased migration in luteal cells (Dickinson et al., 2008). In ovarian development, the SLIT-ROBO signaling pathway may be involved in controlling germ cell migration. Gene expression of SLIT2, SLIT3, ROBO1, ROBO2, and ROBO4 was detected in the ovine fetal ovary at days 50, 60, 70, and 80 of gestation, the time when follicles are formed. In addition, at the time of increased SLIT-ROBO expression, there was a significant reduction in the proliferation of oocytes in the developing ovary (Dickinson et al., 2010). Although GWAS showed that the ROBO1 (BovineHD0100007707) and SLIT3 (BovineHD2000000133) genes are associated with HFC in Nelore, the influence of the SLIT-ROBO pathway on follicle formation in this breed remains to be confirmed by other functional experiments. Nevertheless, this opens a new field for studies on how and whether the SLIT-ROBO pathway affects AFC in Nelore heifers.

A hapFLK study of the Nelore bull genome identified 83,326 SNPs. Most of these are in Chr1, Chr2, Chr5, Chr11, Chr13, and Chr17. The is region in Chr11 is located near genes just between the FSHR and NRXN1 genes. FSHR is required for ovarian follicle development. The authors also highlighted the ‘Roundabout signaling pathway’ involving the ROBO1 and SLIT protein genes (Maiorano et al., 2022). This signaling pathway is important for physiological adaptation of grazing cattle (Muroya et al., 2015).

Two GO terms related to biological processes, kidney development (PKHD1, FBN1, and NID1) and urogenital system development (PKHD1, FBN1, and NID1), were significantly associated with Angus heifers. Fibrillins (FBN1, 2, and 3) are glycoproteins found in connective tissues (Zhang et al., 1995) and form the backbone structure of small diameter microfibrils (Sakai et al., 1991). In addition, fibrillins have regulatory functions by binding and sequestering growth factors. Fibrillin contributes to the extracellular regulation of endogenous TGFB activity by providing a structural platform that controls the diffusion, storage, presentation, and release (Ramirez and Sakai, 2010). In addition, they also interact with the prodomains of bone morphogenic proteins (BMPs) and growth and differentiation factors (GDFs; Sengle et al., 2008), which are members of the TGFB superfamily and have been described as important for ovarian follicular development and function (Dong et al., 1996; McNatty et al., 2000; Yan et al., 2001; Knight and Glister, 2006).

Taurine animals have been shown to have more preantral follicles than indicine animals. On the other hand, indicine animals have more antral follicles counted by ultrasound and histology (Cushman et al., 2019; Favoreto et al., 2019). In addition, follicles from Nelore heifers show higher expression of IGF, BMP, and TGFbeta family genes (Favoreto et al., 2019). These genes are known to be associated with follicle activation and development. The fact that Angus heifers have a polymorphism associated with fibrillin, which regulates these important growth factors, may be one of the reasons for the difference in preantral and antral follicle density in these animals.

Understanding how candidate genes act in modeling the phenotype is a major challenge that is further complicated by the fact that the environment can influence the phenotype. However, the functional information obtained in the present GWAS has focused the study of biological processes and may contribute to future research aimed at validating these genes and elucidating the mechanisms involved in the phenotype. The trait age at first calving in heifers from Nellore exposed to different environmental conditions, especially rainfall and feeding, is associated with regions showing environmental dependence. In animals reared under different environmental conditions, the genomic region on BTA14 acts on metabolic substrates, and these variations are directly involved in the control of reproductive pathways that are essential for early maturity (Mota et al., 2020). BTA 14 also has significant SNPs associated with reproductive function here and in other studies (Kneeland et al., 2004; Buzanskas et al., 2017).

In summary, the GWAS in Nelore and Angus heifers showed SNPs associated with the higher follicle count phenotype. In addition, the HFC heifers had SNPs associated with follicle formation and development.
